# Design of Flexible TPU-Based Lattice Structures for 3D Printing: A Comparative Analysis of Open-Cell Versus Closed-Cell Topologies

**DOI:** 10.3390/polym17091133

**Published:** 2025-04-22

**Authors:** Sergio de la Rosa, Pedro F. Mayuet Ares, Lucía Rodríguez-Parada

**Affiliations:** Department of Mechanical Engineering and Industrial Design, Faculty Engineering, University of Cadiz, 11003 Puerto Real, Cádiz, Spain; pedro.mayuet@uca.es (P.F.M.A.); lucia.rodriguez@uca.es (L.R.-P.)

**Keywords:** lattice structures, TPU, 3D printing, cell topology, elasticity, product design

## Abstract

This study presents a comparative analysis of the influence of open-cell and closed-cell topologies on the manufacturing quality and resultant elasticity of 3D printed thermoplastic polyurethane (TPU) lattice structures. Lattice samples were designed based on various open-cell and closed-cell configurations, varying in unit cell size and fabricated using extrusion-based additive manufacturing (AM) techniques. A microscopic analysis was conducted to assess manufacturing defects, while mechanical compression tests were performed to characterize the elasticity of the samples. The correlation between the obtained results enabled the evaluation of the relationship between the manufacturability of lattice topologies and their stiffness. The findings reveal substantial differences in the manufacturability of the topologies, with open-cell structures exhibiting more pronounced defects. Additionally, the unit cell size and the resulting density of the samples were found to provide design advantages, as closed-cell topologies demonstrated superior load resistance. The accumulation of manufacturing defects was identified as a critical factor influencing deviations in stiffness measurements. This study establishes a foundational framework for lattice structural design, emphasizing the impact of cell topology and unit cell size on mechanical performance. The significance of this research lies in its contribution to the optimization of 3D printed TPU-based lattice structures, providing valuable insights for product design applications.

## 1. Introduction

Additive manufacturing (AM) processes involve layer-by-layer material printing, enabling more active interaction between the properties of manufactured objects and printing variables. This enhances the ability to create highly customized and complex geometries while significantly reducing manufacturing time and costs [1,2]. AM processes have evolved to meet the demand for producing complex structures with good resolution, particularly useful for low-volume production and frequent design changes [1,2]. Also, AM processes have specific advantages and disadvantages resulting in distinct functional and esthetic features. Selective laser sintering (SLS) is commonly used due to its precision and ability to work with a wide range of material powders, including commonly used polymers and elastomers such as thermoplastic elastomers (TPEs), as well as metals like aluminum and titanium. This provides a wide range of properties, ranging from flexibility and elasticity in the case of TPE powders to strength and high-temperature resistance in the case of metal powders. However, SLS printing methods can be expensive and require post-processing. On the other hand, stereolithography (SLA) allows for very small resolutions that are challenging to achieve and may eventually become a practical technique, although compatible resins may not match the strength and mechanical performance of other AM processes [3,4]. In this sense, fused filament fabrication (FFF) arguably represents the most cost-effective explanation for product customization, producing objects with good resolution and mechanical performance at lower manufacturing and material costs [5,6,7,8]. Even so, achieving high-quality prints depends not only on the printing parameters but also on the properties of the material used. In particular, the rheological behavior of the polymer filament is crucial, as it determines how the material flows when heated and its ability to solidify into a stable structure after deposition [8,9]. This rheological behavior directly influences the mechanical flexibility and durability of the final printed object, as it affects layer adhesion, deformation resistance, and overall structural integrity. As a result, one of the main research challenges related to FFF involves understanding the interplay between design and manufacturing variables and their effect on the physical and mechanical properties, as well as the potential for objects’ customization [10,11]. In this context, the growing interest in elastic materials has led to increased research on and development of elastomeric polymers [12,13]. TPEs, and especially thermoplastic polyurethane (TPU), have turned out to be a popular choice among different elastic materials [14,15,16]. TPU-based materials offer great biocompatibility, as well as smoothness that provides a strong and durable adhesion between the printed layers. AM customization capabilities and adaptability to the design process open up new lines of research and application, promoting the interest of these materials for the manufacturing of products with specific elasticity needs [10,17,18]. It is found that the study of the functional properties related to the behavior conferred by the filling material and internal structure is especially relevant [19,20]. It was demonstrated that the filling material and internal structure can play a critical role in defining the global features, making it possible to provide a different elasticity to the same product as well as obtain different elasticities from the same elastic material [13,21,22]. In this sense, TPU, together with the geometric properties of the printed elements, would allow us to obtain specific properties in terms of texture, flexibility, and other sensations [23,24,25], enabling the adjustment of the elasticity in the development of all kinds of 3D printed TPU-based product applications that require a specific elasticity [7,22,26]. Under this pretext, research focused on the functional properties related to the behavior conferred by the filling material and the internal structure [10,27] has greatly expanded the research based on lattice structures [28,29,30,31,32,33,34]. Lattice geometric models are a potential tool for different industrial sectors [35,36,37,38] due to the possibility of adapting and optimizing elastic materials for innovative lightweight and high-performance configurations [39].

Recent research on the compressive behavior of 3D lattice structures at quasi-static loading along with the influence of design and printing parameters has documented a significant effect on the compression properties and deformation mechanisms of lattice samples [40]. A parametric study using experimental and finite element analysis on several lattice designs documented the cyclic compression behavior of TPU, demonstrating that design changes in lattice topology had a notorious impact on the porosity and density of the samples. Increasing porosity was reflected in the increasing compression capacity of the samples due to the greater volume of voids. The strength of lattice samples decreased with increasing porosity, which reduced the reaction forces under the same maximum compressive stress [41]. Also, research related to lattice graded design documented that a rational variation in relative density can significantly enhance the stiffness and energy absorption capability of lattices under high compressive strains [42]. The compressive energy absorption behavior of all structures was dependent on strain rate and cell orientation [43]. The importance of the variation in parameters in the design of reticular structures was validated depending on the variant of elastic material used [43]. In relation to unit cell size parameters, the literature reports that unit cell size plays a critical role among the design parameters. An increase in the unit cell size experimentally led to a reduction in the Young′s modulus of the lattice specimens, decreasing compressive forces under the same maximum deformation [22]. Along with the influence of the angle between struts, the strut diameter, and the number of layers, the literature reports that the angle between struts has a noticeable influence on increasing mean and peak compressive forces [26]. In this sense, unit cell size parameters and lattice topology were established as a viable tool for customizing the elasticity of lattice samples for product design purposes [20,22]. TPU-based lattice samples′ variants and materials were designed and tested in order to be applied in personalized product design [20]. Samples′ elasticity was found to be dependent mainly on parameters such cell topology and the unit cell size of the lattice structure. For structures with different cell topologies, an increase in stiffness could be obtained for the same manufacturing conditions [22]. Despite the growing interest in lattice structures for product design applications, there remains a notable gap in research regarding the fundamental differences between open-cell and closed-cell topologies. Building on previous approaches, this study aims to evaluate the impact of lattice topologies on the elasticity of 3D printed TPU-based lattice samples. Various open-cell and closed-cell topologies are considered, with a focus on unit cell size, to assess both the manufacturing quality and the resulting elasticity of the samples.

To achieve this, lattice samples will be designed and fabricated using extrusion-based AM technologies. Mechanical testing and microscopic analysis will be conducted to characterize the elasticity of the samples. The significance and contribution of this work lie in providing a comprehensive framework for lattice structural design, comparing open-cell and closed-cell topologies, and offering valuable insights for lattice product design applications.

## 2. Materials and Methods

The experiment was proposed with the aim of characterizing the influence of geometric parameters such as unit cell topology and unit cell size on the manufacturing processes and the compressive behavior of the designed lattice samples.

### 2.1. Materials

The material used for the manufacturing of the samples was a 1.75 mm thick commercial TPU, specifically Filaflex 93A filament from Smart Materials 3D company (Alcalá la Real, Jaén, Spain). The mechanical properties given by the manufacturer are detailed in Table 1.

### 2.2. Specimen Preparation

**Figure 1:**
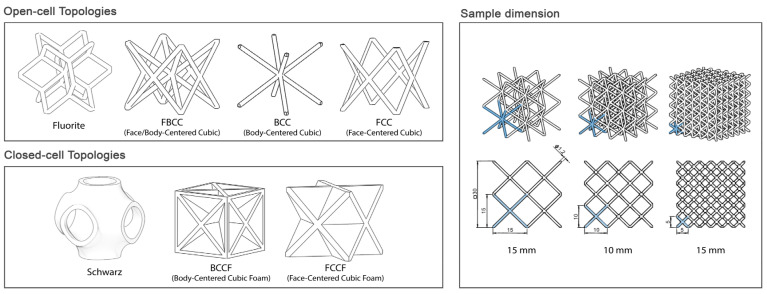
Topologies and sample dimensions.

Designing a closed-cell topology typically involves understanding the functional requirements of an open-cell topology. By closing the openings with thin or thick walls, the open-cell structure can be transformed into a closed-cell configuration while preserving the original functional requirements. In this sense, several open-cell and closed-cell topologies have been designed through generative design and architectural material modules in Ntopology^®^ 3.4 engineering design software. AM principles have been followed for a supportless lattice structure design. Key features such as the overhang angle and bridging distance are critical in creating a support-free lattice structure. The overhang angle is the maximum angle at which a part can be 3D printed without support, with 45° being a safe approximation. Bridging distance depends on the melt viscosity of the extruded material and its cooling rate. Both features can be optimized by adjusting variables such as printing speed and printing temperature [48]. Based on these principles, lattice topologies were selected for their suitability in FFF-based supportless lattice printing. Selected geometries are well established in prior research for their mechanical properties and elasticity, making them ideal for applications in lightweight structures while also ensuring practical relevance in real-world 3D printing applications (Figure 1).

Furthermore, the designed lattice samples were cubic, with a fixed size of 30 mm × 30 mm × 30 mm. The beam thickness for all cell topologies was set to 1.2 mm and three different unit cell sizes were studied in order to characterize the samples’ manufacturability and resulting elasticity.

Samples were modeled using nTopology^®^ 3.4 engineering design software and were processed using the Ultimaker Cura^®^ slicer (Ultimaker B.V., Utrecht, The Netherlands). The equipment used to print the samples was a customized ender 3 pro printer with an indirect extrusion system, a print volume of 220 × 220 × 250 mm and a 50 to 400-micron layer resolution. Several custom elements were included in the FFF machine to ensure print quality for elastic filaments. A Capricorn brand polytetrafluoroethylene (PTFE) tube for the Bowden-type extrusion system was used in order to reduce the coefficient of friction on the filament by using a low-tolerance inner diameter reinforced with high-performance additives. In addition, a customized extruder was used in order to reduce the gap at the entrance hole to the Bowden tube. Based on the preliminary impressions, the printing temperature used was 220 °C due to the reduction in the number of imperfections. On the other hand, the retraction was enabled because of the reduction in the stringing phenomenon. The optimal parameter values selected for the manufacturing process were kept constant for the totality of the samples and are shown in Table 2.

### 2.3. Methods

**Figure 2:**
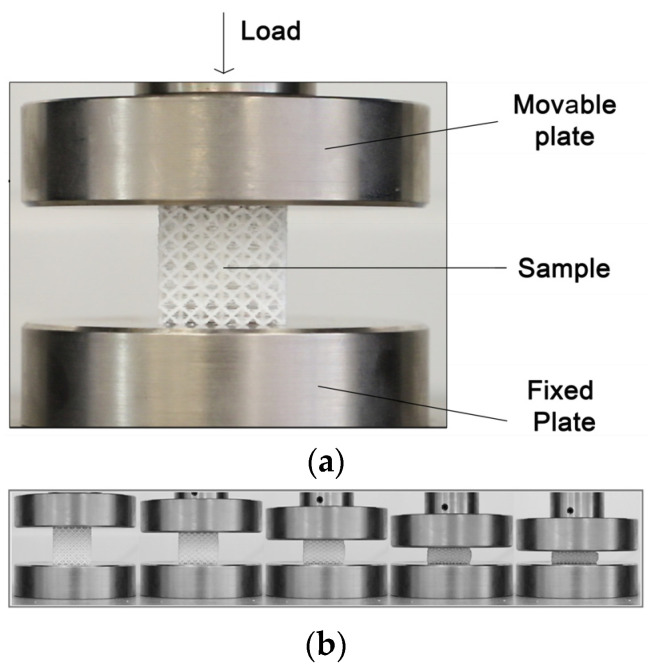
Experimental method: (**a**) set up; (**b**) compression testing.

**Figure A1:**
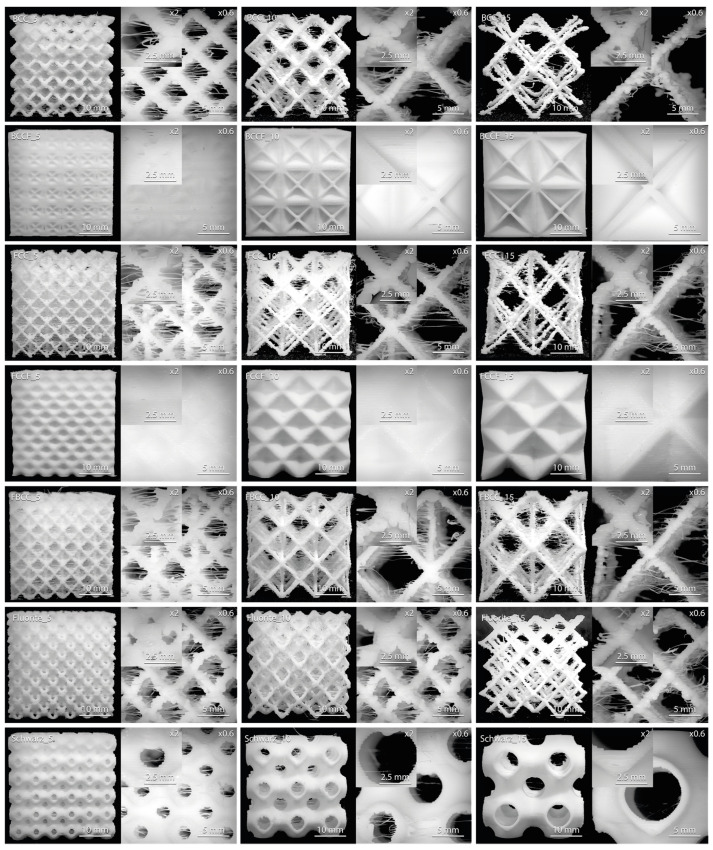
Optic microscopy of printed samples.

Three units of each sample were manufactured in order to consider the average value between the different samples and tests. In this sense, every lattice sample was tested a total of three times. Each of the samples were referenced using the nomenclature. In Appendix A, Figure A1 shows in detail all manufactured lattice samples. This means that a total of 63 samples were manufactured, with a total of 189 compression tests carried out. Compression tests were conducted using a Shimadzu AG-XPLUS (shimadzu, Kyiv, Ukraine) testing machine equipped with a 50 kN load cell capable of performing various static tests, including compression, tension, and bending. The testing machine was managed using Trapezium X software (Simadzu, Kyiv, Ukraine), through which all the variables associated with the sample and those test parameters, such as speed or compression distance, were established. The lattice samples were compressed between the two steel plates of a machine (Figure 2). The lower platen was fixed, while the upper platen moved with constant set speed. The compression speed of the test was set to 1 mm/s, and the total travel distance of the upper plate was set to 20 mm. Consequently, the samples were compressed up to approximately 70% (2/3) of their initial size. This represents a critical deformation threshold where structural collapse or plastic deformation occurs. This value is commonly used to assess energy absorption capacity before total failure, simulating real-world conditions. It also allows for the evaluation of elastic recovery, material densification, and the transition between elastic and plastic behavior in cellular structures. To ensure accurate stress evaluation, individual cross-sectional area measurements were performed for each lattice structure type prior to the commencement of compression testing. This approach addressed the dimensional variability inherent in the diverse lattice structure geometries. By conducting specimen-specific measurements, the assumption of a uniform area was avoided, enabling a more precise stress calculation during data analysis.

All samples were analyzed microscopically using Leica S9i series microscopic equipment (Leica Microsistems S.L.Ul, Wetzlar, Germany) in order to analyze defects derived from the manufacturing process of the samples.

## 3. Results and Discussion

This section shows the results in terms of the influence of cell topology and unit cell size on the sample manufacturing process and the elasticity of elastic lattice structures.

### 3.1. Morphological Analysis

**Figure 3:**
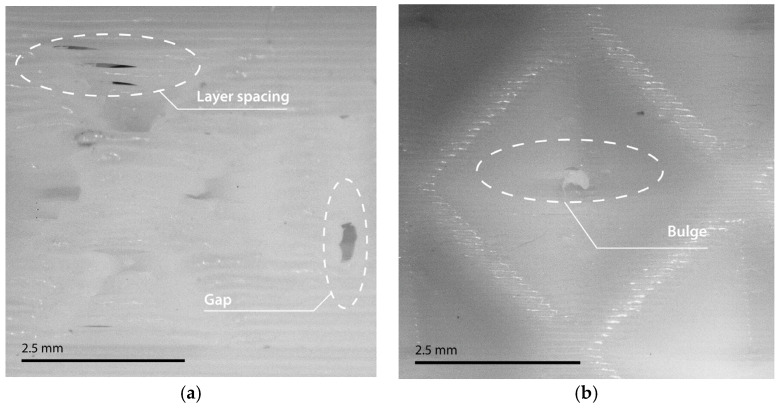

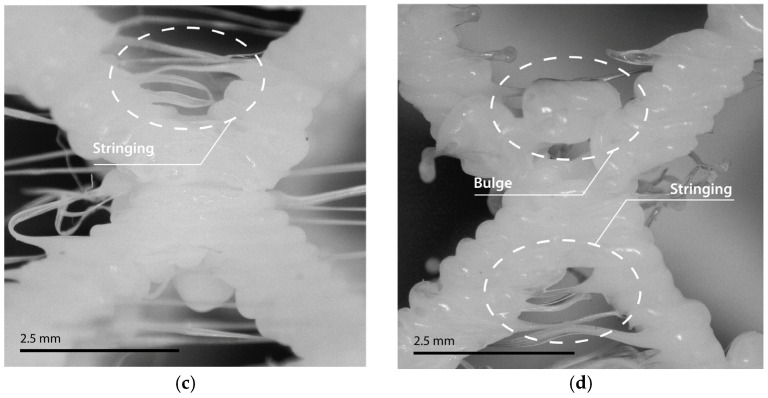
Details of manufacturing defects in samples: (**a**) BCCF_5; (**b**) FCCF_10; (**c**) BCC_5; (**d**) FCC_10.

The printing experience differed depending on the lattice sample. A relevant difference was found between the printing of lattice samples with closed-cell topologies and lattice samples with open-cell topologies. Samples based on open-cell topologies presented poorer manufacturing quality, and the appearance of manufacturing defects was more noticeable with respect to samples based on closed-cell topologies. The main defects found in the optical microscopy analysis are shown in Figure 3. Among the most predominant defects are the layer spacing, material gaps, material strings (stringing), and the formation of material bulges [10,49]. In Appendix A, Figure A1 shows in detail the optical microscopy on the finish and defects of each of the manufactured lattice samples.

It is possible to observe that the mentioned defects occur regardless of the unit cell size or cell topology [50]. However, it was found that quality deficiency was found to be more predominant in open-cell lattice samples in comparison with closed-cell lattice samples. Specifically, the aforementioned quality deficiency was found to be more noticeable in open-cell lattice samples with larger unit cell sizes compared to smaller unit cell sizes where print quality was significantly improved. This phenomenon could be due to the fact that the TPU-based lattice samples can easily deform in contact with the movement of the extruder itself, which would cause buckling and bending issues during the printing [51,52]. In this sense, it was observed that the appearance of the stringing effect and material bulges was more prominent in open-cell lattice samples regardless of the unit cell size. This could be directly related to the increase in the possibility of extruder material dripping when traveling a greater distance without depositing material [53].

However, it was observed that the stringing effect was more aggressive in smaller unit sizes, which could be directly related to the linear increase in the number of retractions of the extruder material with the increase in the number of beams of the lattice samples [52,53]. In another part, material bulges appeared to be slightly more predominant at larger unit cell sizes, which could be entirely related to more aggressive buckling and flexing problems due to the type of lattice structure [51,53]. Closed-cell lattice samples showed no significant differences in print quality regardless of unit cell size. The samples mostly presented layer spacing defects and gaps sporadically on the surface. These defects proved to be more prevalent in structures with larger unit cell sizes. One reason may be the accumulation of defects as a result of the sample having a higher density [10]. In another part, the stringing effect was practically suppressed due to cell topology, appearing slightly at smaller unit sizes of the Schwarz geometry [20].

### 3.2. Sample Compressive Behavior

The printing quality had a direct impact on the compression test and the results obtained. Some of the samples experienced a very slight reaction force due to low structural resistance, causing the machine to suffer some issues in compression force recordings. Stress–strain data obtained from compression tests show typical deformation phases and mechanisms reported by other several authors [21,54]. In the initial stage, an elastic response is observed, where the applied stress is directly proportional to the sample deformation. In the second stage, plastic deformation begins, followed by a progressive plastic collapse (plateau stresses) of the lattice sample cells. In the final stage, the beginning of the densification period where all the cells collapse on each other is shown. The compressive stress “σ” is plotted on the vertical axis, while the strain “ε” is plotted on horizontal axis. “σ” can be obtained from the following equation:

σ = F/S(1)

where F corresponds to the load applied to the sample in the axial direction and S to the surface on which said load is exerted. In the initial stage, for small deformations, samples’ stiffness can be obtained from its equivalence to the slope of the initial elastic region of the stress strain curve using the following equation:

E = dσ/(dε)│ε < εy(2)

where “ε” corresponds to the deformation, which is given by Equation (3):

ε = ∆L/L0(3)

As previously mentioned, compression tests were conducted on a total of three units for each sample. In this regard, Table 3 details the mean stress values and standard deviation for each specimen.

**Figure 4:**
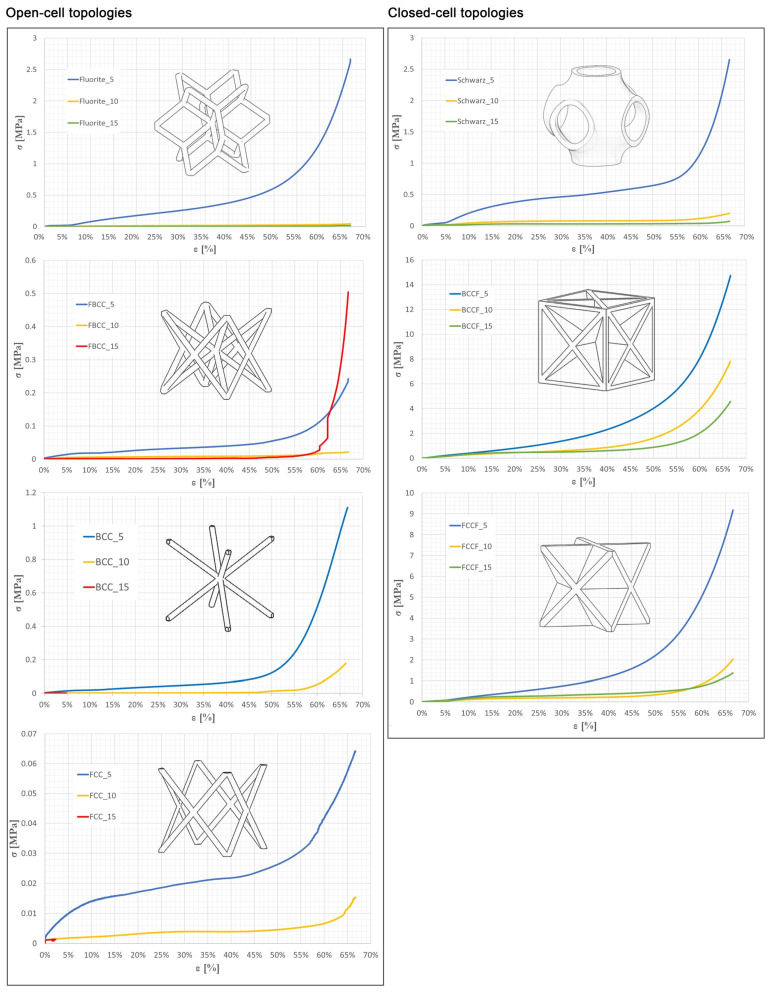
Stress–strain curves of the tested samples.

Subsequently, the results of the average stress–strain curves are presented in Figure 4, where samples exhibiting a minimal reaction force due to low structural resistance are highlighted in red.

Overall, an elastic response was obtained up to approximately 5% deformation. In the same way, plateau stress increased progressively until reaching a deformation of around 50–60% prior to densification. Several effects of sample deformation due to the lattice core were observed. The stress values for each stress–strain curve region were found to be directly related to unit cell sizes and topologies. In general terms, samples with larger unit cell sizes presented higher deformation values compared to samples with smaller unit cell sizes. It was found that the densification period began earlier for samples with smaller unit cell sizes, with approximately 60% deformation for larger unit cell sizes and 50% for smaller unit cell sizes.

**Figure 5:**
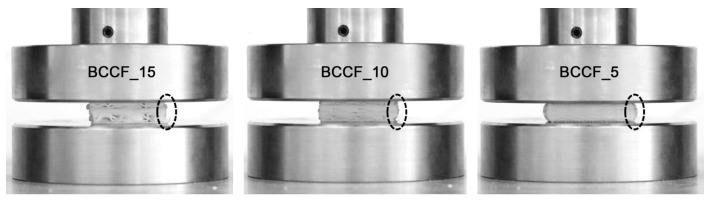
Increase in the barrel effect.

The early onset of the densification period could be related to the unit cell size, since a smaller unit cell size leads to an increase in the number of cells that would cause the cells of the lattice sample to begin to densify more quickly in the compression test. This effect is clearly seen in the barrel effect, where samples with smaller unit size present more noticeably when the moving plate reaches its maximum travel capacity (Figure 5).

In the same way, unit cell sizes and topologies prove to affect collapse and plateau stresses. A clear increase in plateau and densification stresses was found for closed-wall topologies and smaller unit cell sizes, specifically more aggressively for samples with a unit size of 5 mm. This phenomenon could be related to the fact that intrinsically closed-cell lattice samples show design advantages with higher load resistance compared to open-cell lattice structures, and samples with a smaller unit cell size tend to offer better stiffness and buckling resistance [7].

### 3.3. Elasticity Analysis

**Figure 6:**
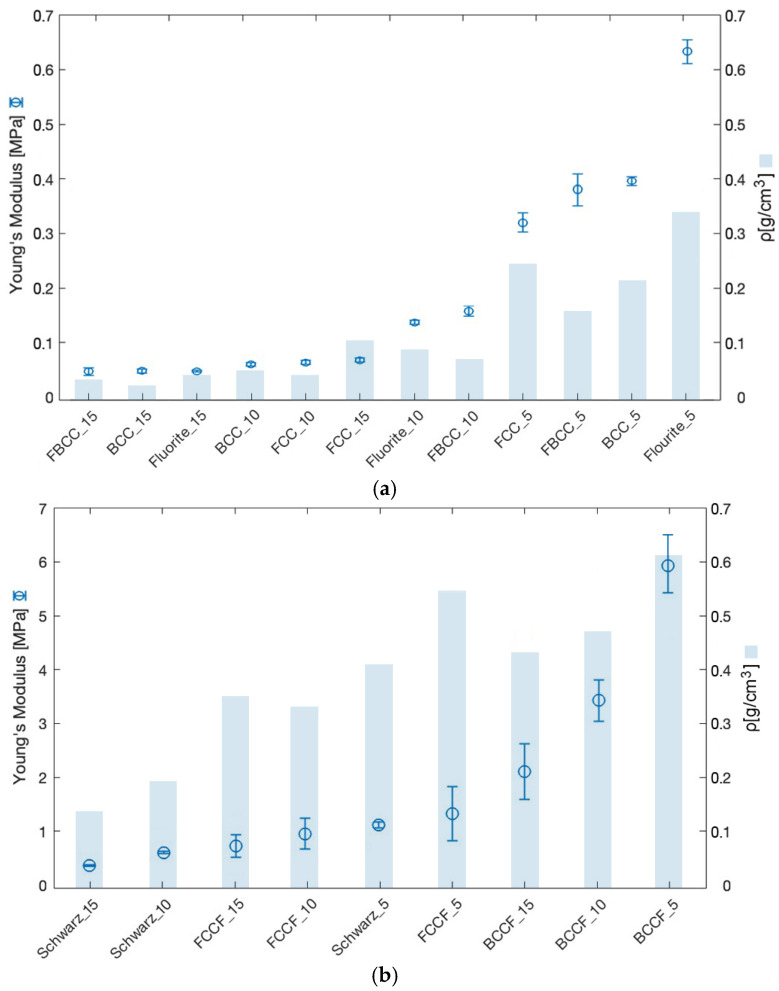
Young’s modulus according to lattice samples: (**a**) open-cell topologies; (**b**) closed-cell topologies.

To determine the lattice samples’ stiffness, the Young′s modulus (E) values of all the samples were obtained from the analysis of the elastic region of the stress–strain curves. Therefore, the range of recorded stresses has been selected between a 0.5 mm and 1.5 mm displacement of the movable plate (equivalent to a deformation between 1.5% and 5%), where the slope of the stress–strain curve showed linear behavior. From the data obtained, a strong correlation was observed between the increase in Young′s modulus with respect to the decrease in unit cell size (Figure 6).

Lattice samples with smaller unit cell sizes presented higher stiffness values regardless of the cell topology. According to [22], this phenomenon could be related to the increase in sample density as a result of the increase in the number of cells. In this sense, the geometric complexity of the sample would be playing a critical role as a consequence of the increase in the number of connections between the beams of the lattice samples. According to [20], both phenomena could directly support the increase in difficulty in compressing the samples, which would explain the progressive increase in the stiffness values obtained. In the same way, the increase in sample stiffness as a function of unit cell size was found to be more aggressive when the unit cell size varied from 15 mm to 10 mm. Analyzing the variation in the number of cells, the samples increase the number of cells from 8 to 27 when the unit cell size decreases from 15 mm to 10 mm. Similarly, the number of cells increases exponentially from 27 to 216 when the unit cell size decreases from 10 mm to 5 mm. As an example, the Young’s modulus of the Schwarz topology increased from 0.484 MPa to 0.556 MPa when the number of cells increased from 8 to 27. However, when the number of cells increased from 27 to 216, the Young’s modulus increased from 0.556 MPa to 1.228 MPa, being a total increase rate of 15% compared to a 120% increase rate.

Analyzing the standard deviation of the samples’ stiffness, a clear trend is observed in increasing deviation for smaller unit cell sizes regardless of the cell topology. According to the morphological analysis discussed in the previous section and according to [39], one of the reasons may be the accumulation of defects as a consequence of sample density. In closed-cell topologies, it was shown that the accumulation of layer spacing defects, bulges, and material gaps is more predominant in smaller units as a consequence of the increase in density. Similarly, in open-cell topologies, the stringing effect and generation of material bulges was shown to be more predominant in smaller unit cell sizes due to the linear increase in the number of extruder material retractions, as well as buckling and bending issues. In both cases, the accumulation of defects could be playing a critical role in the compression process of the samples. This explains why lattice samples offered larger standard deviations in the stiffness value for smaller unit cell sizes.

Similarly, from the perspective of printing defect accumulation, it could be explained why open-cell topologies suffer from a more aggressive variation in stiffness as a function of unit cell size. According to the morphological analysis, it is observed that the closed-cell lattice samples maintain a constant print quality for all unit cell sizes. However, open-cell topologies show structural strength and print quality that varies widely between samples of a larger unit cell size and those of a smaller unit cell size. This phenomenon would justify the more aggressive variation in stiffness for these samples.

Finally, the data obtained showed that closed-cell topologies offer higher stiffness values compared to open-cell topologies. The density-to-stiffness ratio, which represents how much stiffness is achieved relative to the samples’ density, was analyzed to assess the efficiency of lattice topologies. The mean stiffness-to-density ratios for all open-cell topologies was 80%, indicating that stiffness was relatively lower compared to density. In contrast, closed-wall samples exhibited a mean ratio of 30%, suggesting higher stiffness compared to density. In this sense, it was observed that closed-cell topologies are more efficient compared to open-cell topologies. Together with the structural nature of closed topologies, this conclusion would explain why the data obtained globally showed that closed-cell topologies offer higher stiffness values compared to open-cell topologies.

## 4. Conclusions

This study analyzed the influence of lattice topologies on the elasticity and manufacturing quality of 3D printed TPU-based lattice structures. The findings highlight the critical role of unit cell size and topology in print quality and mechanical performance.

Morphological analysis revealed that open-cell topologies exhibited greater manufacturing defects, including material stringing and bulging, particularly at smaller unit cell sizes due to buckling problems and increased extruder retractions. In contrast, closed-cell samples showed minimal variation in print quality, though minor defects were observed in larger unit cell sizes due to density-related accumulations. These findings explain the overall inferior manufacturing quality of open-cell topologies.

Compression testing demonstrated stress values in different deformation stages depending on unit cell size and topology. Closed-cell topologies and smaller unit cells exhibited higher plateau and densification stresses, contributing to increased stiffness and buckling resistance. A greater deviation in stiffness values was observed for smaller unit cells, attributed to defect accumulation, particularly in open-cell structures.

The findings indicated that the efficiency of lattice topologies varies significantly depending on their geometric configuration. Open-cell lattice structures exhibited an average stiffness-to-density ratio of 80%, suggesting that stiffness remains relatively low compared to density. In contrast, closed-wall structures demonstrated a mean ratio of 30%, indicating a higher stiffness relative to density. Different topologies resulted in a wide range of stiffness values, which is of significant interest when selecting a structure for a specific application requiring customized stiffness ranges and particular elastic behavior. Lattice structures with smaller unit cell sizes (5 mm) exhibited greater stiffness and load-bearing capacity, making them ideal for applications requiring structural support and minimal deformability. In contrast, larger unit sizes (10 mm and 15 mm) showed lower loading capacity, making them more suitable for applications that prioritized flexibility and cushioning, such as pressure relief systems and biomedical devices. For instance, fluorite samples with small unit sizes could be considered suitable for applications that demand high strain rates and moderate deformability, while BCCF samples could be deemed ideal for high-impact absorption applications that require substantial energy dissipation. These results provide valuable insights for tailoring lattice structures to specific applications, optimizing mechanical performance for impact absorption, strain rate sensitivity, and customized stiffness requirements in engineering design.

## Figures and Tables

**Table 1:** Filaflex 93A filament properties.

Properties	Unit	Value	Test Method
Density	g/cm^3^	1.21	ISO 1183 [44]
Abrasion resistance	mm^3^	35	ISO 4649 [45]
Shore hardness	Shore A	93	ISO 868 [46]
Tensile strength	MPa	40	ISO 37 [47]

**Table 2:** Main manufacturing parameters.

Properties	Unit	Value
Layer height	mm	0.2
Layer width	mm	0.6
Printing temperature	°C	220
Bed temperature	°C	50
Printing speed	mm/s	10
Displacement speed	mm/s	180
Retraction	-	Enabled
Retraction distance	mm	6.5
Retraction speed	mm/s	40

**Table 3:** Mean stress values and standard deviation.

Specimen	Density (g/cm^3^)	σ Avg (MPa)	Standard Deviation
BCC_15	0.025	0.001	0.0001
BCC_10	0.055	0.012	0.0023
BCC_5	0.209	0.136	0.0448
BCCF_15	0.434	0.803	0.0208
BCCF_10	0.470	1.321	0.0308
BCCF_5	0.621	3.052	0.5612
FBCC_15	0.035	0.004	0.0027
FBCC_10	0.070	0.010	0.0007
FBCC_5	0.168	0.049	0.0046
FCC_15	0.346	0.002	0.0011
FCC_10	0.327	0.004	0.0002
FCC_5	0.543	0.026	0.0009
FCCF_15	0.108	0.429	0.0368
FCCF_10	0.059	0.341	0.0293
FCCF_5	0.239	1.427	0.1123
Fluorite_15	0.051	0.004	0.0002
Fluorite_10	0.099	0.021	0.0037
Fluorite_5	0.347	0.557	0.0113
Schwarz_15	0.133	0.036	0.0023

## Data Availability

The original contributions presented in this study are included in the article. Further inquiries can be directed to the corresponding author(s).
